# Management of Subsequent Pregnancy After Perinatal Death: Results from the UNSURENESS Study

**DOI:** 10.3390/jcm14165748

**Published:** 2025-08-14

**Authors:** Claudia Ravaldi, Laura Mosconi, Greta Cancellieri, Martina Caglioni, Alfredo Vannacci

**Affiliations:** 1 PeaRL Perinatal Research Laboratory, CiaoLapo Foundation, Department of Neurosciences, Psychology, Drug Research and Child Health, University of Florence, 50139 Florence, Italy; claudia.ravaldi@gmail.com (C.R.); laura.mosconi@unifi.it (L.M.); 2 Department of Medicine and Surgery, University Vita-Salute San Raffaele, 20132 Milan, Italy; g.cancellieri@studenti.unisr.it (G.C.); caglioni.martina@hsr.it (M.C.); 3 Department of Neurosciences, Psychology, Drug Research and Child Health, University of Florence, 50139 Florence, Italy

**Keywords:** perinatal loss, pregnancy after stillbirth, healthcare professionals, training needs, communication, shared decision making, continuity of care, multidisciplinary care, trauma-informed care, Italy

## Abstract

**Background:** Pregnancies following perinatal loss present emotional and clinical challenges that require individualized care. While awareness of the psychological vulnerability of bereaved parents is increasing, the perspectives and preparedness of healthcare professionals (HCPs) are still under-investigated. **Methods:** The UNSURENESS study involved a national cross-sectional web-based survey conducted between August 2023 and February 2024. The questionnaire explored sociodemographic data, professional experience, training in perinatal loss care, communication approaches, and clinical decision making. **Results:** Two hundred female HCPs participated (midwives 78.0%). One-third had received specific training on managing pregnancies after perinatal loss. On a 0–4 Likert scale, participants emphasized the importance of addressing the previous loss (M = 3.82; SD = 0.03) and building a trusting relationship with parents (M = 3.78; SD = 0.04). Providing comprehensive information (M = 3.59; SD = 0.05) and promoting shared decision making (M = 3.72; SD = 0.04) followed closely. The most challenging tasks included responding to parental fears (M = 3.37; SD = 0.75) and offering reassurance (M = 3.06; SD = 1.06). Clinical decisions were primarily driven by continuity of care (M = 3.70; SD = 0.56) and parental preferences (M = 3.63; SD = 0.57), rather than national guidelines (M = 3.16; SD = 0.82) or research evidence (M = 2.86; SD = 0.94). **Conclusions:** HCPs are aware of the psychosocial complexity involved in these pregnancies but often lack specific training. There is a clear need for trauma-informed interventions and national guidelines to better support both professionals and bereaved families.

## 1. Introduction

Pregnancy care following stillbirth and neonatal death remains a significantly under-addressed issue in international literature, despite its global prevalence and profound emotional, psychological, and clinical implications. Each year, approximately 2 million families experience a stillbirth, and a comparable number endure the death of a neonate within the first 28 days of life [1,2]. In Italy, the reported incidence of stillbirth is 2.4 per 1000 births, while neonatal death affects 1.7 per 1000 [3]. Notably, over half of bereaved parents conceive again within a year of the loss [4], embarking on a subsequent pregnancy that is often fraught with heightened emotional vulnerability, clinical complexity, and enduring grief.

This transitional period is characterized by a persistent co-occurrence of grief, anxiety, and anticipatory hope. Several studies have underscored how the psychological burden of a prior perinatal loss can resurface or intensify during a new pregnancy, contributing to symptoms of depression, perinatal anxiety, and post-traumatic stress [5,6,7]. These experiences are further compounded by the uncertainty surrounding outcomes and the constant reactivation of the prior trauma during medical appointments and perinatal milestones.

Healthcare professionals (HCPs) play a central role in supporting families through these subsequent pregnancies. However, existing evidence suggests that many parents report negative experiences, citing inconsistent emotional support, inadequate communication, and insufficient acknowledgment of their previous loss by care providers [8]. The quality and continuity of care, as well as the attitudes and preparedness of the clinical team, strongly influence parents’ experiences and psychological wellbeing throughout the perinatal period.

Some national guidelines have begun to respond to these needs. For instance, the Irish Health Service Executive includes explicit recommendations for bereavement care during pregnancies following loss, highlighting the necessity for multidisciplinary teams with specific training in perinatal grief and trauma-informed care [9]. Evidence supports the efficacy of scheduled follow-up visits, consistent clinical care, and individualized psychosocial support in enhancing parental coping and promoting emotional regulation [10,11,12].

Despite these advances, training in bereavement care remains insufficient among many HCPs, leading to uncertainty in managing the psychosocial needs of bereaved families [13,14]. Recent analyses suggest that psychological aspects of care during pregnancy, birth, and the postnatal period are often overlooked, particularly in the context of previous perinatal loss [15]. Yet these aspects are central to the WHO’s Nurturing Care Framework, which emphasizes the critical importance of responsive care, emotional support, and secure relationships in early life as determinants of lifelong health and development [16].

In Italy, in February 2023, a set of recommendations was issued by national scientific societies in obstetrics and gynecology, aiming to promote appropriate care for pregnancies after perinatal loss [17]. These recommendations, grounded in international guidelines and evidence-based practices, advocate for continuity of care, individualized emotional support, and multidisciplinary collaboration. However, they currently hold the status of professional guidance and do not constitute binding national protocols. As such, their implementation remains heterogeneous, and many healthcare settings have yet to formally integrate them into routine clinical practice. In this context, improving the competencies of HCPs and ensuring continuity and integration of care is essential. Understanding how HCPs perceive their role and how they manage these pregnancies is a crucial step toward improving care pathways.

The UNSURENESS (sUpportiNg subSeqUent pREgnaNcy after pErinatal loSS) study was developed to explore these dimensions, with the aim of investigating the practices, perceptions, and training experiences of Italian HCPs involved in the care of pregnancies following stillbirth or neonatal death. By mapping current practices and identifying gaps in support, this study aims to inform future training programs and care models capable of responding more effectively to the complex needs of bereaved families.

## 2. Materials and Methods

The UNSURENESS study was an anonymous web-based, mixed methods and cross-sectional study hosted on the Qualtrics platform (Qualtrics, Provo, UT, USA, www.qualtrics.com) provided by Florence University PeaRL laboratory and was distributed through the social media channels of CiaoLapo Foundation, an Italian charity for perinatal loss support, from 1 August 2023 to 28 February 2024. All data were collected and analyzed anonymously. The survey is provided in the Supporting Information (Supplementary S1).

Participants were considered eligible to complete the survey if they were HCPs involved in care during pregnancy after a perinatal loss. The survey was voluntary and anonymous, no personal data were recorded, and in no way was it possible to identify the individual respondents. Informed consent was provided at the start of the survey once participants had read the participant information and met the eligibility criteria.

The survey consisted of questions across several key areas, including (1) sociodemographic information; (2) knowledge and experience about care during pregnancies after perinatal loss; (3) communication skills; (4) training about caring pregnancies after perinatal loss; (5) clinical management of pregnancies after perinatal loss.

The survey was based on the recommendations of the World Health Organization about “Intrapartum care for a positive childbirth experience” (World Health Organization, 2018). Specifically, these recommendations were taken into account to explore HCPs’ communication skills and knowledge about the care of pregnant couples after a perinatal loss.

### Statistical Analysis and Data Presentation

Survey responses were downloaded and extracted from the online survey tool Qualtrics and imported into Stata BE 18 (StataCorp, College Station, TX, USA) for statistical analysis. Incomplete records were excluded, and descriptive statistics were used to analyze quantitative data. Categorical data were reported as frequencies and percentages and compared using the chi-squared test, whereas continuous data were reported as mean values with standard deviations (SD) or as median [quartiles] and compared using *t*-test or Kruskal–Wallis and Mann–Witney tests. All results were considered to be statistically significant at *p* < 0.05. A mixed-method analysis was conducted on open-end questions with the help of artificial intelligence (AI) as described by Ravaldi et al. (2023) [18,19]. The analysis followed an inductive logic, combining AI-assisted thematic categorization with full manual verification. AI outputs were independently reviewed and refined by two researchers (CR and LM), who manually coded and classified responses using MAXQDA 2018. Discrepancies were discussed and resolved through consensus. This approach ensured methodological rigor and consistency, while enabling the efficient organization of a large volume of qualitative data. Further details on this process are provided in the Supporting Information (Supplementary S2). Graphs were plotted using Tableau Desktop 2024.3 (Tableau Software LLC., San Francisco, CA, USA) or Stata BE 18 (StataCorp, College Station, TX, USA).

## 3. Results

### 3.1. Characteristics of the Sample

The sample consisted of 200 female HCPs; the vast majority of the respondents were midwives (78.0%), followed by doctors (13.0%), nurses (6.0%), and psychologists (3.0%); the majority were from Northern Italy (70.5%), with an average age of 38.9 y (SD 8.9, range 22–68 y) and an average number of years of work of 3.5 y (SD 9.1 years, range 1–38 years). A sensitivity analysis was performed by comparing each professional category separately and by grouping midwives (*n* = 156) versus all other healthcare professionals combined (*n* = 44). In both approaches, no statistically significant differences were observed for any questionnaire item (all *p* > 0.05). Given the absence of measurable differences and considering that these professional categories share the same working environment, clinical experiences, and team-based management of subsequent pregnancies after loss, all respondents were analyzed together despite the predominance of midwives in the sample. The full characteristics of the sample are reported in Table 1.

### 3.2. Professional Experience

Table 2 shows that 88.2% of respondents usually referred their patients to centers for high-risk pregnancies, while only 4.3% did not, and 4.8% are unaware of such centers in their area.

The vast majority of respondents (92.0%) have managed cases of perinatal deaths, with a quarter of them having assisted in more than 10 cases. Regarding subsequent pregnancies after perinatal loss, 13.9% of HCPs have never dealt with such cases, 26.2% have managed fewer than 3 cases, 26.7% have managed 3–5 cases, and 17.6% more than 10 cases. Additionally, 73.8% only managed the labor and birth of women with a history of perinatal loss, without having seen them during the pregnancy. When asked if they were routinely involved in debriefing sessions, 30.5% answered that they had participated in such activities and rated their helpfulness with an average of 3.6 out of 4 (SD 0.5). Debriefing sessions were considered very useful by HCPs who never attended them as well (mean 3.6, SD 0.6). About one-third of the sample (32.8%) underwent specific formal training on managing pregnancies after a loss. This training was rated as highly important (scale 0–4) by both HCPs who experienced it (mean 3.7, SD 0.7) and those who did not (mean 3.6, SD 0.5).

### 3.3. Management of Pregnancies After a Loss

#### 3.3.1. Multidisciplinarity

The sample reported engaging in multidisciplinary discussions when managing pregnancies after perinatal loss; 51.3% discussed cases primarily with colleagues from the same professional group, while 48.7% consulted with professionals from different backgrounds. Midwives and nurses most frequently involved other midwives, obstetricians/gynecologists, and psychologists (77.8%), whereas physicians were more likely to consult only with fellow doctors or, less frequently, with psychologists (18.5%).

Overall, a high degree of multidisciplinary collaboration was confirmed. A large majority of participants (93.1%) stated they never manage these pregnancies alone but always work within teams. The most frequent collaborations were with high-risk pregnancy clinics (50.0%) and obstetric pathology departments (45.4%), followed by birthing room teams (48.1%) and obstetric ward teams (37.1%). To a lesser extent, HCPs also interacted with outpatient services (24.1%) and territorial healthcare providers (19.4%) and more occasionally with specialized clinic teams (11.1%).

Psychological support was available in slightly more than half of the reported cases; 30.6% of HCPs collaborated with in-hospital perinatal psychology services, while 27.8% reported working with territorial psychologists, suggesting that, while integrated psychological support is frequently present, it is not yet universally embedded in multidisciplinary care pathways.

#### 3.3.2. Shared Decision Making

**Figure 1:**
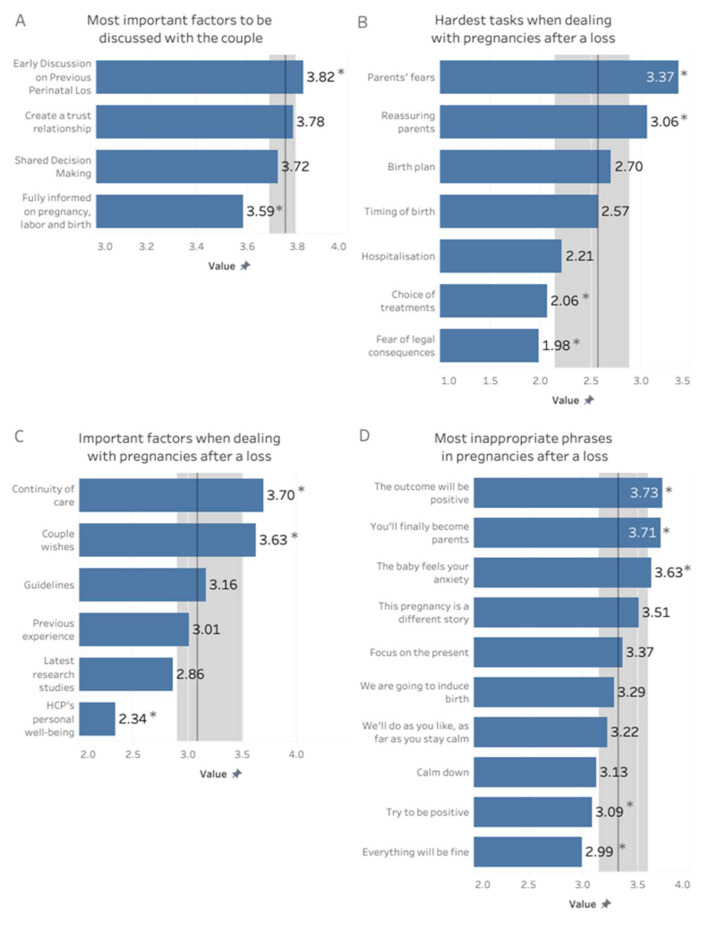
Panel (**A**): Most important factors to be discussed with the couple. Panel (**B**): Hardest tasks when dealing with pregnancies after a loss. Panel (**C**): Important factors when dealing with pregnancies after a loss. Panel (**D**): Most inappropriate phrases in pregnancies after a loss. Grey line: median; shaded area: quartiles; * *p* < 0.01.

Figure 1A shows that HCPs placed slightly more emphasis on discussing previous loss and on establishing trust with parents compared to a shared decision-making approach about clinical management and giving thorough information about care pathways. On a scale 0 to 4, initial discussions on previous losses scored 3.82 (SD 0.03), followed by building a trust relationship (3.78, SD 0.04), higher than providing a shared decision making (3.72, SD 0.04, *p* < 0.05); and ensuring parents were fully informed about pregnancy, labor, and birth is rated significantly lower (3.59 SD = 0.05, *p* < 0.01).

Consistently, HCPs reported that dealing with parents’ fears (3.37, SD 0.75) and reassuring them (3.06, SD 1.06) are considered significantly more difficult tasks than others (*p* < 0.01). Instead, as shown in Figure 1B, technical tasks, such as managing the need for hospitalization (2.21, SD 1.14), avoiding legal issues (1.98, SD 1.09), and setting an appropriate therapy (2.06, 1.00), are perceived as less challenging (*p* < 0.01).

#### 3.3.3. Clinical Management of Pregnancies After a Loss

Concerning the most relevant factors on clinical management of pregnancies after loss, Figure 1C shows that the most highly rated elements by HCPs were continuity of care (3.70, SD 0.56) and respecting the couple’s wishes (3.63, SD 0.57), both of which are considered significantly more important than other factors (*p* < 0.01). Guidelines (3.16, SD 0.82) and previous experience (3.01, SD 0.77), as well as latest research studies (2.86, SD 0.94), are also important, though slightly less emphasized, while the personal well-being of HCPs (mean = 2.34, SD = 0.92) is rated the least important factor (*p* < 0.01).

A summary of prenatal tests and procedures performed in pregnancies after a loss is reported in Table 3. Prenatal care practices reported by HCPs revealed a high adherence to standard protocols for clinical surveillance during pregnancy after perinatal loss. Routine procedures—such as serial blood tests, ultrasound scans, and vital sign monitoring—were widely implemented across all trimesters. Additionally, targeted assessments including nuchal translucency in the first trimester, glucose testing in the second, and group B streptococcus screening in the third were consistently reported. A smaller proportion of providers recommended invasive diagnostics (e.g., chorionic villus sampling or amniocentesis), in line with specific indications. The data confirm that, in the Italian context, pregnancies after loss are managed with intensified clinical surveillance consistent with high-risk protocols, though not necessarily tailored to the emotional or psychological needs associated with bereavement. Prenatal classes were also rated as important by most HCPs (mean 3.2 SD 0.9, on a 0–4 scale).

Table 4 lists information offered by trimester. In the absence of clinical reasons related to the health of the baby or mother, 65.4% of respondents indicated that their facility rarely or never hospitalizes women a few days before birth, while only 18.2% do so often or always. The vast majority of HCPs (86.9%) reported that it would be very useful to have specific national guidelines on the management of pregnancies after a loss.

#### 3.3.4. Labor and Birth Management

Regarding techniques for managing labor in women with previous perinatal loss, the majority of HCPs reported regularly using position changes (75.5%) and free positions (74.5%). Creating a comfortable environment was also widely practiced, with 71.6% indicating regular use. Massage was frequently employed by 62.7% of respondents, followed by warm water techniques at 52.9% and voice use at 49.0%. Music was the least commonly utilized technique, with 43.1% of the sample reporting its regular use.

When it comes to the use of epidural analgesia, 3.3% of HCPs always recommend it, 90.2% discussed it with the couple, and 6.5% had other approaches. In the absence of clinical indications for known pathologies, 70.7% of HCPs recommend waiting for spontaneous labor, 20.7% suggested induction, and 8.7% had other recommendations. The perceived influence of the delivery room environment on labor progress was rated at an average of 3.5 (SD = 0.72), and its influence on how the couple remembers the labor was rated at an average of 3.55 (SD = 0.67).

HCPs rated the importance of involving the partner in various phases of labor and birth at an average of 3.76 (SD = 0.52) on a scale from 0 (not at all) to 4 (extremely). Additionally, the perceived helpfulness of the partner is rated at an average of 3.79 (SD = 0.46) on the same scale.

#### 3.3.5. Information and Psychological Aspects

HCPs rated the importance of providing clear and comprehensible information about various aspects of care during pregnancies following perinatal loss at an average of 3.7 (SD = 0.47), significantly higher than the rating for the usefulness of providing written information to couples, which averaged 3.3 (SD = 0.80; *p* < 0.0001).

Regarding the features of the relationship between HCPs and parents, 8.7% of respondents considered it “difficult”, 41.3% find it engaging, and 46.7% find it extremely engaging. When considering psychological aspects such as postpartum depression, 67.4% of HCPs believe it affects 30% of women with a previous perinatal loss, while 23.9% estimate it affects 70%, and 8.7% estimate it affects 12%. Regarding perinatal anxiety, 97.8% of HCPs report it to be more frequent in women with a previous perinatal loss, while only 1.1% believe it to be the same or less frequent. After the birth of the subsequent child, 55.4% of HCPs observe that anxiety does not reduce, 18.5% believe it does, and 26.1% are unsure. In terms of grief processing, 63.0% of HCPs believe it can slow down or worsen with a new pregnancy, 19.6% think it remains the same, 16.3% are unsure, and only 1.1% believe it concludes.

We asked HCPs how they rated their preparedness in supporting couples through the psychological aspects of a pregnancy following a perinatal loss, only 1.0% of HCPs feel very prepared, 12.5% feel adequately prepared, 41.3% feel uncertain, 38.5% feel poorly prepared, and 6.7% feel not prepared at all. Consistently, the vast majority of the sample strongly (27.9%) or fully supports (70.2%) the promotion of comprehensive training to enhance HCPs’ psychological support skills.

Respondents rated various phrases for couples dealing with a pregnancy following perinatal loss on a scale from 0 (very useful) to 4 (very inappropriate). The most inappropriate phrases included “This pregnancy can only have a positive outcome” (rated 3–4 by 97.1%), “Now, finally, you are also about to become parents” (94.3%), “Calm down, because the baby feels your worry” (93.3%), “This is a different story, let’s not make hasty comparisons” (89.4%), “The past is past, we must focus on the present” (82.7%), “Be positive” (82.7%), “We will do as you wish, as long as you stay calm” (80.8%), “We will induce labor earlier, for safety” (80.7%), “Stay calm” (78.9%), and “Everything will be fine” (71.1%). Figure 1D shows mean values of inappropriateness of each phrase.

#### 3.3.6. Easy and Difficult Aspects in Assisting Pregnancies After Perinatal Loss

HCPs were asked to identify the easiest and most difficult aspects of assisting pregnancies following a perinatal loss. The responses were analyzed through an AI-assisted thematic analysis (see [18] for methodological details) providing insight into both the supportive and challenging elements of their roles.

Easier Aspects of Care

Several HCPs reported that establishing a trusting relationship with the couple and offering emotional presence were among the most manageable tasks. Commonly mentioned elements included psychological support, welcoming attitude, listening, attention, empathy, emotional understanding, and being present. Clear communication (explaining every step, giving positive news), professional confidence (clinical knowledge, certainty of one’s competencies), and teamwork (working in group, sharing decisions) were also seen as facilitating factors. From a clinical perspective, routine monitoring, conducting regular check-ups, and maintaining physiological birth processes were described as relatively straightforward, as they follow established protocols. HCPs also cited reliable patient attendance, patients’ adherence to medical advice, and the fact that couples often appeared more prepared, informed, or motivated as helpful aspects. The opportunity to share a moment of joy and hope, to witness a rebirth, and to support parents who actively seek connection with their unborn child were perceived as emotionally rewarding. Some professionals emphasized the ease of providing care when psychological support was already in place, or when couples had previously engaged in childbirth education, peer groups, or psychotherapy.

More Difficult Aspects of Care

In contrast, the most frequently reported difficulties concerned managing anxiety, fear, and emotional vulnerability—both of the parents and of the professionals themselves. Reported challenges included maternal anxiety, uncontrolled fear, emotional overload, and the pressure to ensure a positive outcome. The need to constantly reassure, to contain parental anxiety, and to support bonding with the unborn child was described as highly demanding. HCPs also reported difficulty in addressing past trauma, including unprocessed grief, fear of recurrence, and feelings of guilt in the parents. A lack of confidence, institutional guidelines, or adequate training was mentioned as compounding these difficulties. Other recurring issues were the challenge of providing individualized psychological support, navigating emotionally intense communication, and helping parents integrate the previous loss with the new experience. Further difficulties included decision making around timing and mode of birth, especially when influenced by emotional distress rather than clinical indications. Supporting the couple through ambivalence, past memories, and fear of another loss, while maintaining professional calmness and empathy, was identified as particularly complex.

In summary, while technical and procedural aspects were often perceived as manageable, the emotional and relational demands of supporting pregnancies after loss emerged as the most taxing for HCPs, requiring sustained emotional labor and sensitivity.

## 4. Discussion

It is well established that perinatal loss may have profound and enduring consequences on parents’ mental health and overall life trajectory [14,20]. In this context, a subsequent pregnancy often constitutes a period of psychological overload, where the fear of reliving the trauma of loss permeates every aspect of the experience [12,21]. This pervasive anxiety may eclipse moments of hope and joy typically associated with gestation, leading to a heightened state of emotional ambivalence and hypervigilance [22]. For both mothers and their partners, the new pregnancy entails coping simultaneously with the demands of a new life and the unresolved grief of the previous loss—an emotionally complex situation that requires careful, sustained, and empathetic support from HCPs [12].

However, findings from our study suggest that such support is not always attuned to parents’ actual needs. The prevailing care model reported by respondents was characterized by a predominantly paternalistic approach, with limited involvement of parents in clinical decision making. Rather than facilitating shared dialogue and meaning making, HCPs often prioritized reassurance as the primary communicative response. As highlighted in recent literature, this tendency to reassure may reflect the clinician’s discomfort with emotional suffering rather than the patient’s expressed needs [23]. Although intended to provide relief, reassurance offered prematurely or without acknowledgment of the underlying distress can inadvertently silence grief and hinder authentic emotional connection. In a context where clinical and emotional uncertainty are both salient, such dynamics may erode trust and compromise the therapeutic alliance between parents and care providers.

In our study, no statistically significant differences emerged when comparing professional categories separately or by grouping midwives versus all other healthcare professionals combined. This supports the decision to analyze the full sample as a whole, consistent with the Italian perinatal care setting, where these professionals share the same working environment, clinical experiences, and team-based management of subsequent pregnancies after loss. Our sample demonstrated a high level of awareness regarding the importance of continuity of care, identifying it as the most relevant factor in the clinical management of pregnancies following perinatal loss. This emphasis reflects an understanding of how continuity facilitates the establishment of trusting relationships between parents and HCPs, while also preventing the emotional fatigue associated with repeatedly narrating the story of the previous loss. The pivotal role of continuity of care in supporting bereaved families has been highlighted in qualitative and retrospective studies, including recent research from the Rainbow Clinic in Manchester, where mothers reported that consistent care relationships helped them feel acknowledged and emotionally safer throughout the pregnancy [24].

Despite this awareness, the UNSURENESS study also brought to light a tendency among HCPs to prioritize clinical needs and patients’ wellbeing over their own psychological and emotional balance. Although the reported clinical management aligns with standard protocols for high-risk pregnancies, our findings suggest that subsequent pregnancies after perinatal loss are generally approached through a biomedical lens, with limited differentiation from other obstetric risk categories. While this vigilance is crucial from a medical standpoint, it may not sufficiently address the specific psychosocial complexities and trauma-related needs of bereaved parents. The frequent application of intensive surveillance tools (e.g., serial ultrasounds, frequent blood testing, and fetal monitoring) likely serves a dual purpose—medical assessment and emotional reassurance. However, the lack of distinct protocols or individualized pathways specifically tailored to parents with a history of perinatal loss reflects a potential gap between clinical routine and trauma-informed care models recommended in international guidelines. Integrating emotional, relational, and psychological dimensions—beyond standard surveillance—remains an area for improvement within the multidisciplinary care framework. While such behavior may stem from a deep sense of professional responsibility, it also risks increasing the likelihood of emotional exhaustion and burnout. This is particularly concerning in the context of perinatal bereavement care, where HCPs are frequently exposed to emotionally intense narratives and existential suffering. Previous studies have shown that providers working closely with bereaved families are at elevated risk of compassion fatigue and burnout symptoms, especially in the absence of adequate psychological support and reflective practices [25,26].

Indeed, psychological support also emerged as a key area of concern among HCPs in this study, yet it continues to receive less attention than biomedical aspects of care. As previously discussed, parents navigating a new pregnancy after loss are often emotionally fragile and may struggle to connect with the unborn child. Campbell-Jackson et al. (2014) [7] describe how the experience of a prior loss can lead parents to emotionally distance themselves from the fetus during the prenatal period, driven by anticipatory grief and fear of reliving trauma. Conversely, when the child is born alive, some mothers report a sense of emotional numbness that impedes immediate bonding, requiring time and support to adjust to the new reality [5,7].

Taken together, these findings underscore the urgent need for psychological care to be embedded as a routine component of clinical pathways—not only during pregnancy but continuing through the postnatal period and into early parenthood. Integrating mental health support up to the child’s second year of life aligns with the WHO’s Nurturing Care Framework, which emphasizes the foundational role of responsive caregiving and parental mental health in early child development [27]. Such an approach would not only improve outcomes for families but could also mitigate emotional burden among HCPs through shared responsibility and structured support systems.

With regard to HCPs’ knowledge of the incidence of anxiety and depressive symptoms during pregnancies following perinatal loss, our findings revealed a degree of uncertainty among respondents. This lack of clarity may have practical repercussions, including difficulties in recognizing and responding appropriately to emerging symptoms, and a tendency either to overestimate or underestimate the psychological burden experienced by parents. This aligns with recent Italian research indicating that HCPs—particularly midwives—often lack sufficient knowledge about perinatal mental health, especially in the context of bereavement and psychological vulnerability [15]. The identified gap underlines the critical need for structured training pathways that equip professionals with both theoretical knowledge and clinical tools to assess and manage psychological distress in perinatal care settings.

Encouragingly, our participants expressed a clear awareness of the need to implement dedicated training on psychological support, emphasizing the value of relational competence in effectively responding to parents’ needs. Indeed, such skills are not ancillary but central to perinatal care in contexts of trauma and grief. Communication strategies and psychological sensitivity form the backbone of a trauma-informed approach to maternity care, which is increasingly recognized as essential in preventing re-traumatization and promoting emotional safety [28]. This approach advocates for care environments that are attuned to the lived experiences of patients, acknowledging the potential impact of prior trauma on the perinatal experience and adapting clinical practices accordingly.

Effective communication is particularly crucial in this setting; the way in which information is conveyed can either support recovery or reinforce distress. As Heazell et al. (2024) argue, language that minimizes or invalidates parental emotions—even unintentionally—can disrupt the therapeutic alliance and contribute to cumulative psychological harm [11]. Although HCPs in our sample demonstrated high awareness of inappropriate communicative practices, a non-negligible proportion (approximately 20–30%) still reported the use of phrases such as “keep calm” or “everything will be fine.” These expressions, although commonly intended as comforting, may be perceived as dismissive or invalidating in the context of a prior loss, again reflecting a reassurance-driven dynamic that favors emotional containment over authentic validation.

The analysis of HCPs’ experiences in supporting pregnancies following a perinatal loss offers important insights into both the rewarding and challenging dimensions of their roles. Empathy and emotional presence emerged as relatively manageable aspects of care, underscoring the deep human connection inherent in perinatal practice. The ability to express empathy, actively listen, provide psychological support, and engage in clear communication was perceived—also in the qualitative responses—as a natural and accessible component of care. Several HCPs specifically referred to listening, being present, creating a bond, and supporting parents as core facilitators in establishing trust and alleviating emotional distress. Routine clinical activities such as monitoring fetal wellbeing and conducting regular check-ups were also commonly cited as easier aspects, due to their standardized nature and familiarity within obstetric care.

However, this perceived ease in showing empathy contrasts with the difficulty reported by HCPs in providing reassurance—often interpreted as a necessary counterpart to emotional support. As outlined by the American Psychiatric Association, empathy involves understanding another person’s experience from their own perspective and does not inherently require corrective or soothing responses [29]. Reassurance, by contrast, is frequently driven by the clinician’s discomfort with distress rather than the patient’s actual needs and may risk obscuring the parent’s lived experience. In our analysis, managing fear and anxiety, responding to unspoken grief, and supporting bonding with the unborn baby were among the most frequently mentioned challenges. Additional difficulties included the lack of specific psychological protocols, the absence of structured training, and the need to contain the parents’ emotional fragility while maintaining clinical decision making. This dynamic suggests a potential confusion between empathy and emotional management and highlights the need for deeper training on the nuances of empathic care. Future studies could explore how HCPs conceptualize empathy in clinical settings, in order to inform the development of more targeted training strategies.

Teamwork and interdisciplinary collaboration were also identified as central facilitators of high-quality care. Working within a multiprofessional team was perceived as beneficial, enabling a more comprehensive and responsive approach to the multifaceted needs of bereaved families. These findings are consistent with international evidence emphasizing the value of coordinated team-based care in tailoring support to the unique and evolving needs of parents following perinatal loss [11].

### Strengths and Limitations

The UNSURENESS study provides one of the first comprehensive explorations of HCPs’ perspectives on managing pregnancies following perinatal loss in the Italian context. One of its main strengths lies in the inclusion of a large, diverse sample of 200 female HCPs, including midwives, physicians, nurses, and psychologists from various regions of Italy. The mixed-methods approach—combining quantitative and qualitative data—allowed for a nuanced analysis of both practice patterns and emotional dimensions of care. Furthermore, the survey tool was explicitly grounded in internationally validated frameworks, such as the WHO’s Intrapartum Care for a Positive Childbirth Experience and the Nurturing Care Framework, ensuring conceptual consistency and policy relevance.

Nonetheless, this study is subject to several limitations. First, as with all voluntary online surveys, there is a risk of self-selection and response bias. Professionals with a particular interest or prior experience in bereavement care may have been more motivated to participate, potentially limiting the generalizability of the findings and leading inaccurate conclusions. Second, all respondents were female, which—while reflective of the gender distribution in Italian perinatal care—does not allow for the exploration of gender-based differences in attitudes or approaches. For this reason, our findings may have limitations in their generalizability to a broader population. Moreover, we recognize that the predominance of midwives in our sample and the limited size of other professional groups may have prevented the detection of more subtle differences. Third, the cross-sectional nature of the study precludes any assessment of causality or changes over time in training, attitudes, or practice. Additionally, although the qualitative data offer rich insights, they were generated via open-ended survey questions rather than in-depth interviews or focus groups, which may have limited the depth and spontaneity of the narratives. Future research should aim to address these limitations by employing longitudinal or ethnographic methods, integrating perspectives from multiple stakeholders—including bereaved parents—and expanding the sample to include male healthcare professionals and more varied professional settings. Larger and more balanced samples across professions will allow a more detailed exploration of potential profession-specific perspectives in the Italian context and their comparison with international findings. Moreover, examining organizational factors and institutional culture could yield critical insights into the facilitators and barriers to high-quality care in pregnancies after loss.

## 5. Conclusions

The UNSURENESS study offers a novel and in-depth contribution to understanding the challenges and opportunities in the care of pregnancies following perinatal loss within the Italian healthcare system. The findings highlight critical gaps in HCPs’ preparedness, particularly regarding the recognition and management of parental psychological distress, and the consistent application of effective communication and emotional support strategies. These limitations point to the urgent need for structured and evidence-based training programs that can equip HCPs with relational and trauma-informed skills.

Capturing the lived experiences and perceived needs of healthcare providers is a foundational step toward the development of national guidelines capable of ensuring respectful, equitable, and psychologically attuned care for bereaved families. The integration of trauma-informed principles and the WHO’s Nurturing Care Framework across the entire continuum of care—from the time of loss to the postnatal period of the subsequent pregnancy—emerges as a promising direction to mitigate emotional suffering and support healthy parent–infant relationships.

Furthermore, future research should explore the conceptualizations of empathy and reassurance held by HCPs, in order to foster a paradigm shift from a reactive, problem-solving approach toward one grounded in presence, emotional containment, and relational continuity. Such a transformation is essential for promoting a care model that not only manages clinical risks but also sustains parents through the emotional complexity of navigating pregnancy after loss.

## Figures and Tables

**Table 1:** Characteristics of the sample.

		*n*	%
Geographical Zone	North	141	70.5%
	Center	36	18.0%
	South	23	11.5%
Age Class	<35 y	80	40.0%
	36–42 y	63	31.5%
	≥43 y	57	28.5%
Years of Work	<8 y	74	37.0%
	9–16 y	60	30.0%
	≥17 y	66	33.0%
Job	Midwife	156	78.0%
	Doctor	26	13.0%
	Nurse	12	6.0%
	Psychologist	6	3.0%
		200	100.0%

**Table 2:** Professional experience.

		*N* (%)/ Mean (SD)
* N *		200 (100.0%)
Presence of a center for at-risk pregnancies		
	Yes, I refer women there	165 (88.2%)
	Yes, I don’t refer women there	8 (4.3%)
	No	5 (2.7%)
	Don’t know	9 (4.8%)
Perinatal deaths assisted		
	None	15 (8.0%)
	<3	33 (17.6%)
	3–5	46 (24.6%)
	6–10	47 (25.1%)
	>10	46 (24.6%)
Subsequent pregnancies assisted		
	None	26 (13.9%)
	<3	49 (26.2%)
	3–5	50 (26.7%)
	6–10	29 (15.5%)
	>10	33 (17.6%)
Assisted only labor, not pregnancy		
	Yes	138 (73.8%)
	No	37 (19.8%)
	Don’t remember	12 (6.4%)
Debriefing experience available		
	Yes	57 (30.5%)
	No	122 (65.2%)
	Don’t know	8 (4.3%)
Debriefing experience importance (0–4)		
Available and attended		3.6 (0.5)
Not available/never attended		3.6 (0.6)
Specific training on perinatal loss		
	Yes	59 (32.8%)
	No	119 (66.1%)
	Don’t remember	2 (1.1%)
Usefulness of training (0–4)		3.6 (0.6)
Attended		3.7 (0.7)
Never attended		3.6 (0.5)

**Table 3:** Prenatal tests and procedures performed in pregnancies after a loss.

Test/Procedure	* N * (%)
1st trimester	
Vital signs	95 (81.9%)
Urine test	102 (87.9%)
Urine culture	96 (82.8%)
Blood test	113 (97.4%)
Ultrasound	113 (97.4%)
Pap test	44 (37.9%)
Nuchal translucency	104 (89.7%)
Chorionic villus sampling	19 (16.4%)
Amniocentesis	15 (12.9%)
2nd trimester	
Blood test	112 (96.6%)
Vital signs	109 (94.0%)
Fundal height	97 (83.6%)
Glucose test	82 (70.7%)
Ultrasound	115 (99.1%)
3rd trimester	
Blood test	115 (99.1%)
Vital signs	110 (94.8%)
Fundal height	105 (90.5%)
Strep test	106 (91.4%)
Anti-d prophylaxis	110 (94.8%)

**Table 4:** Information offered by trimester for pregnancies after a loss.

Information Provided	I Trimester	II Trimester	III Trimester	Post-Partum	Never
Lifestyle counseling	95 (88.0%)	3 (2.8%)	1 (0.9%)	3 (2.8%)	6 (5.6%)
Psychological wellbeing	84 (77.8%)	3 (2.8%)	4 (3.7%)	5 (4.6%)	12 (11.1%)
Nurturing care	26 (24.1%)	20 (18.5%)	34 (31.5%)	20 (18.5%)	8 (7.4%)
Labor	3 (2.8%)	29 (26.9%)	69 (63.9%)	1 (0.9%)	6 (5.6%)
Birth mode	7 (6.5%)	35 (32.4%)	57 (52.8%)	1 (0.9%)	8 (7.4%)
Pain management	1 (0.9%)	23 (21.3%)	72 (66.7%)	1 (0.9%)	11 (10.2%)
Hospital contacts	6 (5.6%)	21 (19.4%)	73 (67.6%)	1 (0.9%)	7 (6.5%)
Breastfeeding	1 (0.9%)	17 (15.7%)	65 (60.2%)	13 (12.0%)	12 (11.1%)
Psychological support	69 (63.9%)	15 (13.9%)	11 (10.2%)	4 (3.7%)	9 (8.3%)
Prenatal classes	36 (33.3%)	55 (50.9%)	6 (5.6%)	2 (1.9%)	9 (8.3%)
Cephalic presentation	5 (4.6%)	33 (30.6%)	59 (54.6%)	1 (0.9%)	10 (9.3%)
Postpartum services	16 (14.8%)	7 (6.5%)	53 (49.1%)	20 (18.5%)	12 (11.1%)

## Data Availability

The data that support the findings of this study are available from the corresponding author upon reasonable request.

## Supplementary Materials

The following supporting information can be downloaded at: https://www.mdpi.com/article/10.3390/jcm14165748/s1.
