# Supplementary material for: Management of Subsequent Pregnancy After Perinatal Death: Results from the UNSURENESS Study
Source: J Clin Med. 2025 Aug 14;14(16):5748. doi: 10.3390/jcm14165748 (PMC12386835; doi:10.3390/jcm14165748)
Supplement: Supplementary file 1 [file jcm-14-05748-s001.zip › jcm-3776205 Supplementary S1 - AI assisted qualitative analysis.pdf]

AI-assisted qualitative analysis in the UNSURENESS Study

The qualitative analysis conducted in the UNSURENESS study follows a methodological framework previously validated in a larger dataset[1]. That study applied AI-assisted thematic analysis to a dataset of 1,774 responses, totaling over 59,000 words, to explore emotional and experiential patterns in perinatal care. The present study adapted this approach to a smaller set of open-ended responses collected from healthcare professionals (HCPs) regarding lactation support following perinatal loss. The smaller sample size allowed for a more detailed manual review while preserving the efficiency provided by AI-assisted thematic identification. The process was structured in multiple phases, beginning with data preprocessing, followed by AI-assisted thematic detection, and culminating in a manual validation and classification phase.

Data preprocessing involved minimal modifications to maintain the natural linguistic structure of responses. Basic anonymization procedures were applied, along with text cleaning steps such as standardizing capitalization, removing extraneous punctuation, and addressing minor typographical inconsistencies. Unlike quantitative responses that rely on predefined categories, open-ended text requires a flexible yet structured approach to thematic identification. To facilitate this, an AI-assisted step was used as an initial filter, allowing for systematic organization of the data while maintaining contextual relevance.

The AI model employed in this phase was trained on a dataset specific to perinatal loss and lactation, ensuring domain-appropriate categorization. The model's role was limited to suggesting thematic clusters and identifying potential connections between responses that may not have been immediately evident through manual coding alone. The AI-generated output consisted of a set of proposed themes, each accompanied by a confidence score indicating the likelihood of alignment with a given category. This preliminary classification provided the foundation for subsequent manual verification.

Following AI-based categorization, all responses were imported into MAXQDA 2018 for manual review. Two independent researchers (CR and LM) manually examined the AI-generated classifications to validate, refine, or reassign themes as necessary. This iterative verification ensured that AI-suggested categories accurately reflected the context and intent of respondents' statements. Discrepancies between AI outputs and human interpretations were resolved through systematic review and discussion. The process also involved merging overlapping themes, eliminating redundant classifications, and consolidating subthemes into broader thematic groups. Given the smaller dataset of the LISTEN study compared to the 2023 validation study, full manual verification of all responses was feasible.

To quantify the prevalence of identified themes, thematic frequency distributions were calculated, determining which topics were most frequently mentioned by HCPs. This provided a quantitative perspective on qualitative findings, highlighting the most commonly reported challenges and identifying areas where additional training or institutional support may be needed. This step was particularly critical for assessing patterns across different professional roles and levels of experience.

To refine the quantitative evaluation of themes, the methodological framework outlined in Ravaldi et al. (2023)[1] was expanded using techniques we described in a recent study on AI-assisted keyword-based analysis[2]. In brief, keyword-based thematic analysis was enhanced using ChatGPT (OpenAI 2024) and Claude 3.5 Sonnet (Anthropic 2024), which were independently employed to extract and classify relevant keywords from responses. These two AI models were used to generate and expand

the keyword list, ensuring comprehensive coverage of linguistic variations and professional terminology. Open-ended survey answers were then scanned for the presence of these keywords, and each response was assigned to one or more themes based on keyword occurrence.
To validate the robustness of the AI-assisted thematic detection, keyword assignment was repeated independently using both ChatGPT and Claude, and their results were compared. The two AI-generated classifications achieved full concordance in keyword identification, confirming the reliability of thematic categorization. This approach mitigated potential model-specific biases and ensured that detected themes were consistent across different AI frameworks.

References
1 Ravaldi C, Mosconi L, Bonaiuti R & Vannacci A. The Emotional Landscape of Pregnancy and Postpartum during the COVID-19 Pandemic in Italy: A Mixed-Method Analysis Using Artificial Intelligence. Journal of Clinical Medicine. 2023 12 (19): 6140. https://doi.org/10.3390/jcm12196140.

2 Ravaldi C, Vannacci A. Small hands, big ideas: exploring nurturing care through Beatrice Alemagna's 'What is a Child?'. Med Humanit. 2025 medhum-2025-013226. doi: 10.1136/medhum-2025-013226.
