# Supplementary material for: Management of Subsequent Pregnancy After Perinatal Death: Results from the UNSURENESS Study
Source: J Clin Med. 2025 Aug 14;14(16):5748. doi: 10.3390/jcm14165748 (PMC12386835; doi:10.3390/jcm14165748)
Supplement: Supplementary file 1 [file jcm-14-05748-s001.zip › jcm-3776205 Supplementary S2 survey.pdf]

Sezione 1 – Dati socio-demografici e professionali	Section 1 – Socio-demographic and professional data
1. **Anno di nascita** (numerico)	1. **Year of birth** (numeric)
2. **Genere**	2. **Gender**
○ Maschio	○ Male
○ Femmina	○ Female
○ Preferisco non rispondere	○ Prefer not to say
3. **Regione in cui lavora** (menu a tendina con elenco delle regioni italiane)	3. **Region where you work** (drop-down list of Italian regions)
4. **Provincia in cui lavora** (menu a tendina con elenco delle province italiane)	4. **Province where you work** (drop-down list of Italian provinces)
5. **Tipo di struttura in cui lavora principalmente**	5. **Type of facility where you mainly work**
○ Ospedale pubblico	○ Public hospital
○ Ospedale/clinica privata	○ Private hospital/clinic
○ Consultorio/servizi territoriali	○ Community health centre/territorial services
○ Altro (specificare)	○ Other (please specify)
6. **Qualifica professionale**	6. **Job title**
○ Medico	○ Physician
○ Ostetrica/o	○ Midwife
○ Infermiera/o	○ Nurse
○ Psicologa/o	○ Psychologist
○ Altro (specificare)	○ Other (please specify)
7. **Anni di esperienza professionale** (valore intero)	7. **Years of professional experience** (integer value)
8. **Livello più alto di formazione professionale**	8. **Highest level of professional training**
○ Laurea triennale	○ Undergraduate degree
○ Specializzazione post-laurea	○ Postgraduate specialization
○ Laurea magistrale	○ Master’s degree
○ Dottorato di ricerca (PhD)	○ PhD
○ Altro (specificare)	○ Other (please specify)

Sezione 2 – Esperienza professionale con il lutto perinatale

9. **Ha mai assistito una donna/coppia che ha vissuto un lutto perinatale?**
   - o Sì
   - o No
10. **Se sì, in quale/i contesto/i?** *(risposta multipla)*
    - o Aborto spontaneo
    - o Morte in utero
    - o Morte neonatale
    - o Interruzione di gravidanza per malformazione fetale
    - o Altro (specificare)
11. **Quanti casi di lutto perinatale ha seguito nella sua carriera?** (numerico)
12. **Ha ricevuto una formazione specifica sull'assistenza al lutto perinatale?**
    - o Sì, durante gli studi universitari
    - o Sì, durante la specializzazione post-laurea
    - o Sì, attraverso formazione continua/aggiornamento professionale
    - o No
13. **Se sì, specifichi il tipo di formazione ricevuta** (campo aperto)

Section 2 – Professional experience with perinatal loss

9. **Have you ever provided care to a woman/couple who experienced perinatal loss?**
   - o Yes
   - o No
10. **If yes, in what context(s)?** *(multiple choice)*
    - o Miscarriage
    - o Stillbirth
    - o Neonatal death
    - o Termination of pregnancy for fetal anomaly
    - o Other (please specify)
11. **How many cases of perinatal loss have you been involved in over the course of your career?** (numeric)
12. **Have you received specific training on bereavement care?**
    - o Yes, during undergraduate studies
    - o Yes, during postgraduate studies/specialization
    - o Yes, through continuing education/professional development
    - o No
13. **If yes, specify the type of training received** (open text)

Sezione 3 – Assistenza alle gravidanze successive a un lutto perinatale

Scala di frequenza:
1 = Mai
2 = Raramente
3 = Qualche volta
4 = Spesso
5 = Sempre

14. **Ha mai assistito una donna/coppia durante una gravidanza successiva a un lutto perinatale?**
    - Sì
    - No
15. **Nella sua esperienza, quanto spesso le gravidanze successive a un lutto vengono gestite con…** *(Scala di frequenza)*
    - Maggior monitoraggio clinico rispetto alle gravidanze fisiologiche
    - Maggior supporto psicosociale rispetto alle gravidanze fisiologiche
    - Continuità assistenziale con lo stesso team per tutta la gravidanza
    - Coinvolgimento di professionisti della salute mentale
    - Colloqui specifici dedicati alla perdita precedente

Section 3 – Care of subsequent pregnancies after perinatal loss

Frequency scale:
1 = Never
2 = Rarely
3 = Sometimes
4 = Often
5 = Always

14. **Have you ever been involved in the care of a woman/couple during a pregnancy following a perinatal loss?**
    - Yes
    - No
15. **In your experience, how often are subsequent pregnancies after a loss managed with…** *(Frequency scale)*
    - Increased clinical monitoring compared to physiological pregnancies
    - Increased psychosocial support compared to physiological pregnancies
    - Continuity of care by the same healthcare team throughout pregnancy
    - Involvement of mental health professionals
    - Specific counselling sessions focused on the previous loss

Sezione 4 – Difficoltà percepite e bisogni professionali	Section 4 – Perceived difficulties and professional needs
**Scala di sicurezza:**<br>1 = Per niente sicuro/a<br>2 = Poco sicuro/a<br>3 = Moderatamente sicuro/a<br>4 = Abbastanza sicuro/a<br>5 = Completamente sicuro/a<br><br>16. **Quando assiste una gravidanza successiva a un lutto perinatale, quanto si sente sicuro/a nei seguenti ambiti?** *(Scala di sicurezza)*<br>o Gestione clinica<br>o Comunicazione con i genitori<br>o Offerta di supporto emotivo<br>o Collaborazione con altri professionisti<br>o Gestione delle proprie emozioni<br><br>17. **Quali tra i seguenti elementi l'aiuterebbero a sentirsi più sicuro/a nell'assistere queste gravidanze?** *(risposta multipla)*<br>o Ulteriore formazione clinica<br>o Formazione sulle competenze comunicative e relazionali<br>o Accesso a consulenza specialistica<br>o Riunioni multidisciplinari<br>o Linee guida/protocolli istituzionali<br>o Supervisione/peer support<br>o Altro (specificare)	**Confidence scale:**<br>1 = Not at all confident<br>2 = Slightly confident<br>3 = Moderately confident<br>4 = Very confident<br>5 = Completely confident<br><br>16. **When managing a pregnancy after perinatal loss, to what extent do you feel confident in the following areas?** *(Confidence scale)*<br>o Clinical management<br>o Communication with parents<br>o Providing emotional support<br>o Collaboration with other professionals<br>o Managing your own emotions<br><br>17. **Which of the following elements would help you feel more confident in assisting these pregnancies?** *(multiple choice)*<br>o Additional clinical training<br>o Training in communication and relational skills<br>o Access to specialist consultation<br>o Multidisciplinary meetings<br>o Institutional guidelines/protocols<br>o Peer support/supervision<br>o Other (please specify)

Sezione 5 – Coinvolgimento del partner

Scala di utilità:
1 = Per niente utile
2 = Poco utile
3 = Mediamente utile
4 = Abbastanza utile
5 = Molto utile

18. **Nella sua esperienza, quanto spesso il partner è attivamente coinvolto nell'assistenza durante una gravidanza successiva a un lutto perinatale?** *(Scala di frequenza)*
19. **A suo parere, quanto è utile il coinvolgimento del partner per il benessere della coppia in questo contesto?** *(Scala di utilità)*

Section 5 – Partner involvement

Usefulness scale:
1 = Not useful at all
2 = Slightly useful
3 = Moderately useful
4 = Very useful
5 = Extremely useful

18. **In your experience, how often is the partner actively involved in the care during a pregnancy after perinatal loss?** *(Frequency scale)*
19. **In your opinion, how useful is partner involvement for the couple's well-being in this context?** *(Usefulness scale)*

Sezione 6 – Supporto psicologico

20. **Ritiene utile offrire supporto psicologico durante una gravidanza successiva a un lutto perinatale?** *(Scala di utilità)*
21. **Quanto spesso nella sua realtà viene effettivamente offerto il supporto psicologico?** *(Scala di frequenza)*
22. **Quali sono i principali ostacoli all'offerta di supporto psicologico nella sua realtà?** *(risposta multipla)*
    - Mancanza di professionisti
    - Mancanza di tempo
    - Mancata consapevolezza del bisogno
    - Mancanza di supporto istituzionale
    - Altro (specificare)

Section 6 – Psychological support

20. **Do you believe it is useful to offer psychological support during a pregnancy after loss?** *(Usefulness scale)*
21. **How often is psychological support actually offered in your setting?** *(Frequency scale)*
22. **What are the main barriers to offering psychological support in your setting?** *(multiple choice)*
    - Lack of professionals
    - Lack of time
    - Lack of awareness of need
    - Lack of institutional support
    - Other (please specify)

Sezione 7 – Affermazioni sull'assistenza

Scala di accordo:
1 = Per niente d'accordo
2 = Poco d'accordo
3 = Né d'accordo né in disaccordo
4 = Abbastanza d'accordo
5 = Molto d'accordo

23. **Indichi il suo livello di accordo con le seguenti affermazioni riguardo l'assistenza alle gravidanze successive a un lutto perinatale** *(Scala di accordo)*:

    1. Un monitoraggio clinico più frequente è sempre rassicurante per i genitori.
    2. La stessa équipe dovrebbe seguire la donna/coppia per tutta la gravidanza.
    3. Parlare della perdita precedente durante le visite può aumentare l'ansia dei genitori.
    4. È importante coinvolgere il partner in tutte le fasi dell'assistenza.
    5. Il supporto psicologico dovrebbe essere offerto di routine nelle gravidanze successive a un lutto perinatale.
    6. I genitori tendono a sovrastimare il rischio di recidiva.
    7. L'operatore sanitario dovrebbe cercare di ridurre al minimo le paure dei genitori.
    8. È meglio evitare di discutere i dettagli della perdita precedente se non sono i genitori a chiederlo.
    9. Nelle gravidanze successive a un lutto, la medicalizzazione è necessaria per garantire la sicurezza.

Section 7 – Statements about care

Agreement scale:
1 = Strongly disagree
2 = Disagree
3 = Neither agree nor disagree
4 = Agree
5 = Strongly agree

23. **Please indicate your level of agreement with the following statements regarding the care of subsequent pregnancies after perinatal loss** *(Agreement scale)*:

    1. Providing more frequent clinical monitoring is always reassuring for the parents.
    2. The same healthcare team should follow the woman/couple throughout the entire pregnancy.
    3. Talking about the previous loss during antenatal visits can increase parents' anxiety.
    4. It is important to involve the partner at all stages of care.
    5. Psychological support should be routinely offered in pregnancies after perinatal loss.
    6. Parents tend to overestimate the risk of recurrence.
    7. Healthcare professionals should try to minimize the parents' fears as much as possible.
    8. It is advisable to refrain from discussing the details of the previous loss unless the parents request it.
    9. In pregnancies after loss, medicalization is necessary to ensure safety.
    10. The woman's emotional state can influence the progress of the pregnancy.

10. Lo stato emotivo della donna può influenzare l’andamento della gravidanza.	

Sezione 8 – Utilità percepita di alcune frasi

Scala di utilità:
1 = Per niente utile
2 = Poco utile
3 = Mediamente utile
4 = Abbastanza utile
5 = Molto utile

24. **Secondo lei, quanto sono utili le seguenti frasi nell'assistenza a una gravidanza successiva a un lutto perinatale?** *(Scala di utilità)*

- Calmati, perché il bambino sente la tua preoccupazione
- Questa gravidanza può solo avere esito positivo
- Andrà tutto bene
- State tranquilli
- Il passato è passato, bisogna concentrarsi sul presente
- Adesso finalmente anche voi state per diventare genitori
- Lo facciamo nascere prima, per sicurezza
- Facciamo come volete voi, basta che state tranquilli
- Questa è un'altra storia, non facciamo paragoni azzardati

Section 8 – Perceived usefulness of certain sentences

Usefulness scale:
1 = Not useful at all
2 = Slightly useful
3 = Moderately useful
4 = Quite useful
5 = Very useful

24. **In your opinion, how useful are the following sentences when caring for a pregnancy after perinatal loss?** *(Usefulness scale)*

- Calm down, because the baby can feel your worry
- This pregnancy can only have a positive outcome
- Everything will be fine
- Stay calm
- The past is past, we must focus on the present
- Now, at last, you too are about to become parents
- We will deliver earlier, for safety
- We will do as you want, as long as you stay calm
- This is another story, let's not make unfounded comparisons

Sezione 9 – Aspetti percepiti come più facili o più difficili	Section 9 – Aspects perceived as easier or more difficult
25. **Nella sua esperienza, quali sono i tre aspetti più facili nell'assistenza alle gravidanze successive a un lutto perinatale?** Li scriva in ordine di importanza, indicandone almeno uno.<br>● Facile 1: ____________<br>● Facile 2: ____________<br>● Facile 3: ____________<br>26. **Nella sua esperienza, quali sono i tre aspetti più difficili nell'assistenza alle gravidanze successive a un lutto perinatale?** Li scriva in ordine di importanza, indicandone almeno uno.<br>● Difficile 1: ____________<br>● Difficile 2: ____________<br>● Difficile 3: ____________	25. **In your experience, what are the three easiest aspects in the care of pregnancies after a perinatal loss?** Please list them in order of importance, indicating at least one.<br>● Easy 1: ____________<br>● Easy 2: ____________<br>● Easy 3: ____________<br>26. **In your experience, what are the three most difficult aspects in the care of pregnancies after a perinatal loss?** Please list them in order of importance, indicating at least one.<br>● Difficult 1: ____________<br>● Difficult 2: ____________<br>● Difficult 3: ____________
**Sezione 10 – Commenti aggiuntivi**	**Section 10 – Additional comments**
27. **Vuole aggiungere altro?** Può utilizzare questo spazio per aggiungere liberamente qualunque commento, riflessione o informazione desideri (si ricordi di non inserire dati personali che la possano rendere riconoscibile, altrimenti il record verrà cancellato).	27. **Would you like to add anything else?** You may use this space to freely add any comment, reflection, or information you wish (please remember not to include personal data that could identify you, otherwise the record will be deleted).
